# Egr-1 inhibits the expression of extracellular matrix genes in chondrocytes by TNFα-induced MEK/ERK signalling

**DOI:** 10.1186/ar2595

**Published:** 2009-01-14

**Authors:** Jason S Rockel, Suzanne M Bernier, Andrew Leask

**Affiliations:** 1 Canadian Institutes of Health Research Group in Skeletal Development and Remodeling, Schulich School of Medicine & Dentistry, The University of Western Ontario, London, Ontario N6A 5C1, Canada; 2 Department of Anatomy and Cell Biology, Schulich School of Medicine & Dentistry, The University of Western Ontario, London, Ontario N6A 5C1, Canada; 3 Division of Oral Biology, Schulich School of Medicine & Dentistry, The University of Western Ontario, London, Ontario N6A 5C1, Canada

## Abstract

**Introduction:**

TNFα is increased in the synovial fluid of patients with rheumatoid arthritis and osteoarthritis. TNFα activates mitogen-activated kinase kinase (MEK)/extracellular regulated kinase (ERK) in chondrocytes; however, the overall functional relevance of MEK/ERK to TNFα-regulated gene expression in chondrocytes is unknown.

**Methods:**

Chondrocytes were treated with TNFα with or without the MEK1/2 inhibitor U0126 for 24 hours. Microarray analysis and real-time PCR analyses were used to identify genes regulated by TNFα in a MEK1/2-dependent fashion. Promoter/reporter, immunoblot, and electrophoretic mobility shift assays were used to identify transcription factors whose activity in response to TNFα was MEK1/2 dependent. Decoy oligodeoxynucleotides bearing consensus transcription factor binding sites were introduced into chondrocytes to determine the functionality of our results.

**Results:**

Approximately 20% of the genes regulated by TNFα in chondrocytes were sensitive to U0126. Transcript regulation of the cartilage-selective matrix genes Col2a1, Agc1 and Hapln1, and of the matrix metalloproteinase genes Mmp-12 and Mmp-9, were U0126 sensitive – whereas regulation of the inflammatory gene macrophage Csf-1 was U0126 insensitive. TNFα-induced regulation of Sox9 and NFκB activity was also U0126 insensitive. Conversely, TNFα-increased early growth response 1 (Egr-1) DNA binding was U0126 sensitive. Transfection of chondrocytes with cognate Egr-1 oligodeoxynucleotides attenuated the ability of TNFα to suppress Col2a1, Agc1 or Hapln1 mRNA expression.

**Conclusions:**

Our results suggest that MEK/ERK and Egr1 are required for TNFα-regulated catabolic and anabolic genes of the cartilage extracellular matrix, and hence may represent potential targets for drug intervention in osteoarthritis or rheumatoid arthritis.

## Introduction

Chondrocytes maintain articular cartilage through coordinated production and degradation of the extracellular matrix. Type II collagen, aggrecan, and link protein – encoded by the genes Col2a1, Agc1 and Hapln1, respectively – are major components of the articular cartilage extracellular matrix (ECM). Type II collagen is the major structural collagen of articular cartilage [1]. Aggrecan is the most abundant proteoglycan, and is responsible for resisting the compressive forces imposed on articulating joints [2]. Finally, link protein stabilizes the association of aggrecan with hyaluronic acid [3]. The expression of these ECM proteins is regulated by transcription factors within the nucleus promoting or inhibiting transcript production. Sry-type high-mobility group box-9 (Sox9) is a regulatory transcription factor that binds DNA at specific sites within Col2a1, Agc1 and Hapln genes to induce their transcription [4-6].

In diseases such as rheumatoid arthritis and osteoarthritis there is a shift in the equilibrium in cartilage production and degradation towards catabolism. TNFα, a potent inflammatory mediator, is found at higher levels in the synovial fluid bathing articular cartilage in diseased joints compared with that of normal, healthy joints [7-9]. Previous work has shown that treatment of chondrocytes with TNFα downregulates the expression of Col2a1, Agc1 and Hapln1 without inducing apoptosis [10-13]. Furthermore, the activation of NFκB) by TNFα signalling reduces Sox9 activity, possibly through competition for the transcriptional cofactor p300 [10,12]. Other signalling pathways are known to be activated by TNFα, however, including the extracellular regulated kinase (ERK)/mitogen-activated protein kinase pathway (reviewed in [14]).

TNFα initiates the activation of ERK/mitogen-activated protein kinase through the adaptor protein, Grb2, binding to the TNFα receptor 1, leading to activation of the ras/mitogen-activated kinase kinase (MEK)/ERK signalling cascade [15]. In immortalized chondrocytes and primary rat chondrocytes, ERK1/2 can be phosphorylated as early as 15 minutes of treatment with TNFα [10,11]. Inhibition of MEK1/2 signalling can attenuate the decreases in Col2a1, Agc1 and Hapln1, as determined by northern blot analysis [10,11]. TNFα also regulates the activity of NFκB and Sox9 in chondrocytes [10,12]. TNFα-induced NFκB DNA binding in immortalized chondrocytes is reduced by inhibition of MEK1/2 signalling [10]. TNFα may therefore regulate the expression of a subset of genes by alterations in the activity of these transcription factors in a MEK1/2-dependent manner.

Although some information is known about selected changes in chondrocyte gene expression in response to TNFα-activated MEK/ERK signalling, the overall impact of this pathway on changes to the chondrocyte gene expression and the downstream transcriptional mechanisms mediating these changes has been poorly defined. We sought to identify the extent to which MEK/ERK may contribute to the overall changes in chondrocyte gene expression in response to TNFα.

In the present study, we found that ERK1/2 undergoes multiple temporal phosphorylation events in response to TNFα-induced MEK1/2 activation. We discovered that approximately 20% of the genes that changed at least 1.45-fold with TNFα were dependent on MEK1/2 activation. A significant subset of these genes encoded proteins that localized to the extracellular space and had collagenase or hyaluronic acid binding activities. We determined that specific matrix metalloproteinases and cartilage-selective ECM transcript levels were regulated by MEK/ERK, while transcripts of the inflammatory gene macrophage colony stimulating factor 1 (Csf-1), were regulated in a MEK1/2-independent manner. Surprisingly, the activation of NFκB and the inhibition of Sox9 activity by TNFα were independent of MEK1/2. The DNA binding activity of the transcription factor early growth response 1 (Egr-1), however, was regulated by TNFα-activated MEK1/2 signalling. Finally, we determined that Egr family members are responsible for the TNFα-induced, MEK-dependent reductions in mRNA transcripts. Egr-1 may therefore regulate a select number of genes in response to TNFα-activated MEK/ERK signalling.

These findings reveal that MEK/ERK-dependent transcription factors that are downstream of TNFα, such as Egr-1, may be targets for therapeutic intervention to treat the pathophysiology of arthritis without disrupting other potential positive effects of TNFα.

## Materials and methods

### Primary chondrocyte culture

Chondrocytes were isolated from the femoral condyles of neonatal (1 day old) rats as previously described [10]. The cartilage canals in newborn rats do not form in the femoral condyles until 5 days postnatal and radiographic signs of the secondary ossification centre do not appear until about 10 days postnatal [16]. Furthermore, to avoid hypertrophic chondrocytes, the upper two-thirds of the cartilage was taken. Cells were plated onto tissue culture plastic (Falcon, Franklin Lakes, NJ, USA) at a density of ~2.5 × 10^4 ^cells/cm^2^. Under these conditions, the culture consists of an essentially pure chondrocyte population.

Monolayer chondrocyte cultures were grown in RPMI 1640 media (Invitrogen, Burlington, ON, Canada) supplemented with 5% foetal bovine serum, 100 U/ml penicillin, 100 μg/ml streptomycin and 1% HEPES buffer (Invitrogen) until approximately 90% confluence was reached (6 to 7 days). Prior to treatment, chondrocytes were incubated in serum-free media overnight. For inhibitor studies, chondrocytes were pretreated with the selective MEK1/2 inhibitor U0126 (10 μM; Promega, Thermo Fisher Scientific, Rockford, IL, USA) [17] for 30 minutes. As previously shown, U0126 has very low inhibitory activity towards other protein kinases [18]. Furthermore, previous studies in our laboratory have demonstrated that 24-hour treatment with 10 μM U0126 had no significant effect on the cell morphology or organization in culture [11]. As controls, cultures were treated in parallel with dimethyl sulfoxide (DMSO) (vehicle for inhibitors), U0124 (10 μM; Calbiochem, EMD Biosciences Inc., La Jolla, CA, USA) or the selective epidermal growth factor receptor inhibitor PD153035 (1 μM; Calbiochem, EMD Biosciences Inc.) [19]. Cultures were then treated with human recombinant TNFα (30 ng/ml; Endogen, Thermo Fisher Scientific) for 15 minutes to 24 hours.

### Antibodies

Antibodies used in this study included anti-phospho-tyrosine-ERK1/2 (E4), anti-Egr-1 (588), anti-α-tubulin (E-19), and anti-NFκB p65 (C-20) antibodies (all from Santa Cruz Biotechnology, Santa Cruz, CA, USA). Horseradish peroxidase-conjugated goat-anti-rabbit or rabbit-anti goat secondary antibodies were obtained from Thermo Fisher Scientific.

### Protein isolation and western blotting

Nuclear and cytoplasmic extracts were isolated using a modified method of Dignam and colleagues [20], as previously described [10]. Total cell extracts were isolated using RIPA buffer as previously described [21]. Protein concentration was determined using the Pierce BCA Protein assay kit (Pierce, Thermo Fisher Scientific), as per the manufacturers' instructions. For western blotting, 20 μg cytoplasmic protein was loaded into 10% polyacrylamide gels containing SDS and separated by electrophoresis. Proteins were transferred onto Protran™ nitrocellulose membranes (Whatman, Inc., Florham Park, NJ, USA) by electroblotting and were stained with Ponceau S to qualitatively determine equal loading of samples and efficient transfer of proteins. Membranes were blocked in 5% nonfat milk (Carnation, North York, ON, Canada) in 0.05% Tris-buffered saline containing 0.05% Tween-20 (TBST) for 1 hour followed by incubation with primary antibodies in blocking buffer overnight. Membranes were washed in TBST and incubated in 5% milk-TBST with appropriate secondary antibody for 45 minutes to 1.5 hours. Membranes were then washed with TBST and rinsed in Tris-buffered saline prior to incubation in Supersignal West Pico Chemiluminescent Substrate (Pierce, Thermo Fisher Scientific) and exposed to Amersham Hyperfilm ECL (GE Healthcare Bio-Sciences Inc., Baie d'Urfé, QC, Canada). Membranes were stripped using 1 M glycine, pH 2.5, and washed using TBST prior to reprobing.

### RNA isolation

Total RNA was isolated from cultures by Trizol (Invitrogen) followed by RNeasy clean-up (Qiagen, Mississauga, ON, Canada) as per the manufacturer's directions. Total RNA was quantified spectrophotometrically. High-quality RNA for use in the microarray analysis was confirmed by analysis in the Agilent 2100 Bioanalyzer (Agilent Technologies, Palo Alto, CA, USA).

### Microarray analysis

Total mRNA (10 μg) from two biological replicates of cells treated with DMSO, U0126, TNFα or U0126 and TNFα, were amplified once and hybridized to RAT230_2.0 gene chips (Affymetrix, Santa Clara, CA, USA). Amplification, labelling, hybridization and detection were performed at the London Regional Genomics Centre (London, ON, Canada) according to the manufacturers' instructions.

### Microarray data and gene ontology analysis

The raw expression values were imported into Genespring GX 7.3 (Agilent Technologies). Raw expression values <0.01 were set to 0.01 and the normalization per chip was set to the 50th percentile. Relative gene expression of the 31,099 probe sets on the chip was determined by normalizing the raw expression values for each probe set to the DMSO control (= one-fold change for each probe set) from each independent experiment. To identify genes that were TNFα-regulated, probe sets that were altered ≥ 1.45 in DMSO/TNFα-treated cultures compared with DMSO-treated cultures were determined for each independent experiment. Probe sets identified as being TNFα regulated in both independent experiments were selected for further analysis. Genes whose transcript levels changed ≥ 1.45-fold were selected for study, as our microarray analysis revealed that aggrecan mRNA – a transcript previously shown to be TNFα sensitive [12] – was reduced approximately 1.45-fold and thus served as a positive control establishing the validity of our microarray data.

To identify probe sets whose changes were altered by TNFα in a MEK1/2-dependent fashion, we normalized the fold change in gene expression of U0126/TNFα-treated cultures to that of cultures treated with U0126 alone from both independent experiments. We determined probe sets that were altered <1.45-fold in response to DMSO/TNFα treatment, and hence were TNFα regulated in a U0126-sensitive fashion. The remainder of the genes on the lists of TNFα-regulated probe sets were determined to be TNFα regulated and MEK independent. Probe sets identified as being TNFα regulated and MEK/ERK dependent or MEK/ERK independent in both independent experiments were selected for further analysis.

Genes were also identified whose basal expression was sensitive to U0126 alone. Probe sets altered ≥ 1.45-fold in response to U0126 treatment relative to DMSO treatment were identified in both independent experiments. The limited number of genes that were altered with U0126 in both experiments (89/31,099) prevented the use of meaningful cluster analysis, but nonetheless served as a potent indication of the selectivity of the U0126 inhibitor. The generated list was then compared with the list of genes changing ≥ 1.45-fold with DMSO/TNFα to identify genes that were basal TNFα independent but MEK/ERK dependent and those genes that were both TNFα and basal MEK/ERK dependent.

The fold change in the transcript levels increased or decreased ≥ 1.45-fold in both independent experiments was averaged. The generated lists of genes determined as TNFα-activated MEK/ERK dependent and TNFα-activated MEK/ERK independent were analysed using the gene ontology browser in Genespring GX 7.3. Major cellular components and molecular functions subcategories of protein products from the list of genes were identified. The resulting list of cellular component ontologies was filtered such that a minimum of 10 genes must be in the initial group of annotated genes from the microarray and the resulting subcategory must be significantly represented (*P *< 0.05). Selected genes within the extracellular space ontology were then organized into subcategories that were significantly represented by the molecular function ontologies (*P *< 0.01).

### Quantitative real-time PCR

Total RNA (25 ng) was amplified using the TaqMan One Step RT-PCR Master Mix (4309169; Applied Biosystems Inc., Streetsville, ON, Canada). Primer/probe sets to rat type II collagen (Col2a1, Rn00564954_m1), aggrecan 1 (Agc1, Rn00573424_m1), link protein (Hapln1, Rn00569884_m1), matrix metalloproteinase-9 (Mmp-9, Rn00579162_m1), matrix metalloproteinase-12 (Mmp-12, Rn00588640_m1), macrophage Csf-1 (Csf-1, Rn00576849_m1) and eukaryotic 18S rRNA (4352930E) were used to analyse relative transcript levels.

Reverse transcription and quantitative real-time PCR reactions were performed using the Prism 7900 HT Sequence Detector (Applied Biosystems Inc.). Samples were incubated at 48°C for 30 minutes to make cDNA templates. The resulting cDNA was amplified for 40 cycles. Cycles alternated between 95°C for 15 seconds and 60°C for 1 minute.

Results were analysed using SDS v2.1 software (Applied Biosystems Inc.). The ΔΔCt method was used to calculate gene expression levels relative to 18S and normalized to vehicle-treated cells. Data were log-transformed prior to analysis by one-way analysis of variance and Tukey's post-hoc test, paired *t *tests and Student's *t *tests, using Graphpad Software v. 4 (Graphpad Software, La Jolla, CA, USA).

### Transfection

Confluent cell cultures were detached using trypsin-ethylenediamine tetraacetic acid (Invitrogen), pelleted, and resuspended in serum-free culture medium. Cells were then plated into 48-well dishes (3.4 × 10^4 ^cells/well) in 200 μl and were transfected with equal amounts of reporter plasmids. The reporter plasmids used in this study included the κB reporter (BD Biosciences, Mississauga, ON, Canada), comprising four tandem repeats of the κB response element upstream of the firefly luciferase reporter sequence and a type II collagen enhancer luciferase reporter (Sox9 reporter) containing four repeats of the 48-base-pair minimal enhancer of the type II collagen gene (pGL3 (4 × 48)) [22]. Each minimal enhancer sequence contains a binding site for Sox9. Multiple repeats of the minimal enhancer are required for optimal firefly luciferase expression [23]. Cells were transfected with 20 μl serum-free media containing the equivalent of 0.156 μg Sox9 reporter or NFκB reporter and 0.352 μl Fugene 6 transfection reagent (Roche Diagnostics Corporation, Indianapolis, IN, USA). In all experiments, chondrocytes were co-transfected with a 0.002 μg renilla luciferase plasmid (pRL-CMV; Thermo Fisher Scientific) to control for transfection efficiency. Cultures were transfected for 4 hours prior to addition of 200 μl foetal bovine serum containing media.

After overnight incubation, the media was aspirated off from the transfected cultures and replaced with serum-free media. Cultures were treated as indicated above and collected using Passive Lysis Buffer (Thermo Fisher Scientific) as directed by the manufacturer. Luciferase activity was measured using the Dual Luciferase Assay System (Thermo Fisher Scientific) in an L-max II microplate reader (Molecular Devices, Sunnyvale, CA, USA). Tanscription-factor-regulated firefly luciferase units were adjusted relative to constitutive cytomegalovirus-regulated renilla luciferase units obtained in control DMSO-treated, U0124-treated or U0126-treated cultures. Data were log-transformed prior to analysis by Student's *t *tests and one-way analysis of variance using Graphpad Software v. 4 (Graphpad Software).

### Electrophoretic mobility shift assays

Binding of nuclear protein complexes to the κB or Egr-1 cognate elements was determined as previously described [10,12]. The double-stranded oligodeoxynucleotides (ODNs) containing the κB cognate sequence (5'-AGTTGAGGGGACTTTCCCAGG-3'), the Egr cognate sequence (5'-GGATCCAGCGGGGGCGAGCGGGGGCGA-3') and the Egr mutant sequence (5'-GGATCCAGCTAGGGCGAGCTAGGGCGA-3') were purchased from Santa Cruz Biotechnology. Competition assays were performed by adding 100-fold molar excess of unlabelled probe to the nuclear extract-labelled probe mixture. Antibody interference assays were performed by adding 2 μg antibody against Egr-1 (specific) or NFκB (nonspecific) 1 hour prior to the addition of nuclear extract to the buffered radiolabelled DNA. Samples were loaded into 4% polyacrylamide gels and were electrophoresed for 3.5 hours. Following electrophoresis, gels were dried and exposed to Amersham Hyperfilm-MP (GE Healthcare Bio-Sciences Inc.) at -80°C.

### Promoter analysis for putative transcription factor binding sites

Upstream regions proximal to the transcriptional start site of the rat Col2a1 and Agc1 genes have been described previously [24,25]. Upstream regions from the transcriptional start site (~5,000 base pairs) of the Rattus Norvegicus Col2a1 [GenBank:NM_012929.1] and Agc1 [GenBank:NM_022190] genes were obtained and analysed for putative transcription factor binding sites by TRANSFAC analysis [26].

### Oligodeoxynucleotide decoy assay

Chondrocytes were plated at 1.2 × 10^6 ^cells/well in six-well culture dishes. Single stranded, phosphorothiol-modified ODNs were annealed by heating complementary ODNs to 98°C for 20 minutes followed by cooling to room temperature for 3 to 4 hours. Chondrocytes were transfected with 2 μM double-stranded ODNs corresponding to the cognate EGR-1 binding sequence (5'-ggaTCCAGCGGGGGCGAGCGGGGgcgA-3') or the Egr mutant sequence (5'-ggaTCCAGCTAGGGCGAGCTAGGgcgA-3'; Sigma Genosys, Oakville, ON, CA) using 1% HiPerfect transfection reagent (Qiagen), as per the manufacturer's instructions. (Lowercase letters indicate phosphorothiol-modified bases.)

To optimize double-stranded ODN transfection conditions, chondrocytes were transfected cells with increasing concentrations of double-stranded, fluorescein-tagged and phosphorothiol-modified ODNs, and the cells were imaged by live-cell fluorescent microscopy (data not shown). Chondrocytes were allowed to grow for 24 hours in the presence of ODNs, after which cells were washed and cultured in serum-free RPMI media overnight. Chondrocytes were treated with TNFα for 24 hours, as described, and total RNA was collected for analysis by real-time PCR.

## Results

### ERK1/2 is phosphorylated by TNFα in chondrocytes

**Figure 1:**
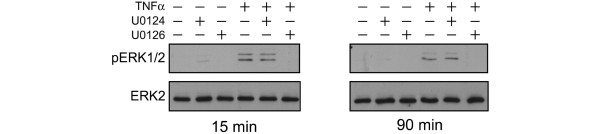
**Multiple ERK1/2 phosphorylation events are dependent on MEK1/2 signalling**. Chondrocytes were pretreated with dimethyl sulfoxide, U0124 (10 μM) or the active mitogen-activated kinase kinase (MEK) 1/2 inhibitor, U0126 (10 μM) for 30 minutes prior to TNFα treatment for 15 minutes or 90 minutes. Cytoplasmic extracts (20 μg) were resolved on 10% polyacrylamide gels and were immunoblotted for pY-extracellular regulated kinase (ERK) 1/2 or total ERK2. Immunoblots are representative of three independent experiments.

We have shown previously that TNFα induces ERK phosphorylation in primary articular chondrocytes 15 minutes post treatment [11]. To confirm and extend these results, we used western blot analysis to show that TNFα induced ERK1/2 phosphorylation 15 minutes post treatment (Figure 1), followed by a decrease in phosphorylation status (data not shown). ERK1/2 phosphorylation was again increased at 90 minutes post treatment (Figure 1). As anticipated, both the increases at 15 minutes and at 90 minutes could be inhibited by the MEK1/2 inhibitor U0126, but not its inactive isoform U0124 (Figure 1). Based on these data, we used U0126 as an inhibitor to assess the effect of blocking MEK1/2 on the mRNA expression pattern modulated by application of TNFα to chondrocytes.

### U0126 blocks part of the TNFα-dependent gene expression changes in chondrocytes

To investigate the global impact of U0126 on TNFα-modulated gene expression in chondrocytes, we utilized microarrays to analyse changes in chondrocyte mRNA expression. Cells were serum-starved overnight and were treated with or without U0126 (10 μM, 30 min) prior to addition of TNFα for 24 hours. Cells were treated with TNFα for 24 hours as previous data showed that this length of TNFα treatment was necessary to generate a TNFα-mediated suppression of chondrocyte matrix genes, owing to the stability of chondrocyte matrix gene mRNAs [27-30].

**Figure 2:**
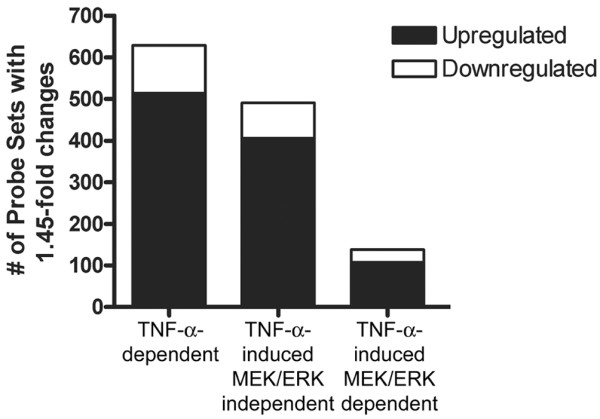
**Activated MEK/ERK signalling regulates a portion of genes regulated by TNFα signalling**. Total mRNA from two independent experiments of primary chondrocytes pretreated with dimethyl sulfoxide or U0126 for 30 minutes followed by treatment with vehicle or TNFα for 24 hours was subjected to microarray analysis. The number of probe sets changing expression ≥ 1.45-fold in TNFα-treated cells (first bar), and the distribution of those changes that are dependent (second bar) or independent (third bar) of mitogen-activated kinase kinase (MEK) 1/2 activity. Probe sets showing ≥ 1.45-fold fold changes with U0126 treatment alone are indicated by the last bar. In order for a probe set to be counted in these categories, the gene needed to be increased or decreased in the same direction ≥ 1.45-fold in both of the two independent experiments. For each bar, the number of genes downregulated (white) and upregulated (black) are indicated. ERK, extracellular regulated kinase.

Microarray analysis from two independent experiments determined that 629 genes were regulated by TNFα signalling in both sets of experiments by at least 1.45-fold, the majority of which were increased in response to TNFα (Figure 2). Of these genes, alterations of 138 (~22%) were attenuated with U0126. Furthermore, of the remaining genes that were not regulated by TNFα, 62 genes were regulated by U0126 alone, indicating that basal MEK/ERK activity may also play a role in chondrocyte gene regulation. Complete microarray data have been deposited in the Gene Expression Omnibus public repository [GEO:GSE14402].

### Selective extracellular matrix and proteinase genes are regulated by TNFα-induced MEK/ERK signalling

We further analysed the lists of genes that were induced by TNFα using specific gene ontologies. Analysis of the list of TNFα-induced, MEK/ERK-dependent and MEK/ERK-independent probe sets indicated that there was significant representation (*P *< 0.05) of genes whose protein products localize to the extracellular space within both lists (Table 1). Further analysis of the list of TNFα-regulated, MEK/ERK-dependent genes – whose products are found in the extracellular space – indicated that some of these genes were significantly categorized by the molecular function of their protein products into categories that included hyaluronic acid binding activity (including Agc1 and Hapln1) and proteinase activity (including Mmp-9 and Mmp-12). Analysis of the TNFα-regulated, MEK/ERK-independent list of genes whose protein products were localized to the extracellular space determined that many of the protein products of these genes were involved in a variety of activities, including chemokine/cytokine activity – including macrophage Csf-1 – and various protease activities. The inflammatory genes, however, appeared to be primarily U0126 insensitive.

**Table 1:** Extracellular space genes regulated at least 1.45-fold by TNFα^a^

Gene	Accession number	Description	Fold change			Basal MEK/ERK dependent
				
			U0126	TNF	TNF + U0126	
**TNFα-regulated MEK/ERK dependent**						
		*Hyaluronic acid binding activity*				
Agc1	[GenBank:NM_022190]	Aggrecan 1	1.14	0.68	1.08	
Hapln1	[GenBank:NM_019189]	Hyaluronan and proteoglycan link protein 1	1.15	0.65	0.96	
		*Collagenase or metallopeptidase activity*				
Mmp-12	[GenBank:NM_053963]	Matrix metallopeptidase 12	0.67	2.92	0.93	
Mmp-9	[GenBank:NM_031055]	Matrix metallopeptidase 9	0.87	1.89	1.01	
		*Metallopeptidase activity*				
Arts1, ERAP1, Appils	[GenBank:NM_030836]	Type 1 TNF receptor shedding aminopeptidase regulator	1.33	1.78	1.55	
		*Complement binding*				
C4bpa	[GenBank:NM_012516]	Complement component 4 binding protein, alpha	1.01	2.19	1.35	
		*Others*				
Amigo2	[GenBank:NM_182816]	Adhesion molecule with Ig-like domain 2	0.99	1.55	1.26	
Cacna2d3	[GenBank:NM_175595]	Calcium channel, voltage-dependent, α_2_/δ_3 _subunit	0.82	1.56	0.99	
Cd68 (predicted)	[GenBank:NM_001031638]	CD68 antigen	1.01	1.80	1.14	
Cgref1, Cgr11	[GenBank:NM_139087]	Cell growth regulator with EF hand domain 1	1.01	0.61	0.83	
Cyp4b1	[GenBank:NM_016999]	Cytochrome P450, family 4, subfamily b, polypeptide 1	1.71	1.72	1.83	
Gm1960, Cinc2, Cinc-2	[GenBank:NM_138522]	Gene model 1960 (NCBI)	0.94	2.38	1.27	
**TNFα-regulated MEK/ERK independent**						
		*Peptidase activity*				
Adam17; TACE	[GenBank:NM_020306]	A disintegrin and metalloproteinase domain 17 (TNF, alpha, converting enzyme)	0.93	1.64	1.41	
Mmp13	[GenBank:XM_343345]	Matrix metallopeptidase 13	0.33	6.80	1.10	*
Ctsc	[GenBank:NM_017097]	Cathepsin C	1.18	2.84	1.72	
Serpinb2, Pai2a	[GenBank:NM_021696]	Serine (or cysteine) proteinase inhibitor, clade B, member 2	0.75	3.47	1.68	
Plat, tPA, PATISS	[GenBank:NM_013151]	Plasminogen activator, tissue	0.94	3.22	1.56	
Plau, UPAM	[GenBank:NM_013085]	Plasminogen activator, urokinase	1.36	2.23	2.87	
C1s, r-gsp	[GenBank:NM_138900], [GenBank:XM_575664]	Complement component 1, s subcomponent	1.05	1.98	1.88	
Cpxm1 (predicted)	[GenBank:XM_215840]	Carboxypeptidase × 1 (M14 family) (predicted)	1.21	3.11	1.84	
Mmp3	[GenBank:NM_133523]	Matrix metallopeptidase 3	0.36	10.26	3.41	*
		*Chemokine activity*				
Ccl5, Scya5, Rantes	[GenBank:NM_031116]	Chemokine (C-C motif) ligand 5	0.90	4.26	1.37	
Cxcl12, Sdf1	[GenBank:NM_001033882], [GenBank:NM_001033883], [GenBank:NM_022177]	Chemokine (C-X-C motif) ligand 12	0.75	2.17	1.33	
Ccl20, ST38, Scya20	[GenBank:NM_019233]	Chemokine (C-C motif) ligand 20	0.65	12.86	5.82	
Cx3cl1, Cx3c, Scyd1	[GenBank:NM_134455]	Chemokine (C-X3-C motif) ligand 1	0.72	4.41	2.95	
Cxcl1, Gro1, CINC-1	[GenBank:NM_030845]	Chemokine (C-X-C motif) ligand 1	0.55	9.18	3.61	*
Cxcl10, IP-10, Scyb10	[GenBank:NM_139089]	Chemokine (C-X-C motif) ligand 10	0.88	2.40	3.22	
Ccl2, MCP-1, Scya2, Sigje	[GenBank:NM_031530]	Chemokine (C-C motif) ligand 2	0.68	32.14	22.59	
		*Cytokine or growth factor activity*				
Bmp2	[GenBank:NM_017178]	Bone morphogenetic protein 2	0.89	1.61	1.53	
Gdf10	[GenBank:NM_024375]	Growth differentiation factor 10	0.59	0.30	0.21	*
Csf1	[GenBank:NM_023981]	Macrophage colony-stimulating factor 1	0.86	2.37	2.17	
Ifngr1	[GenBank:NM_053783]	IFNγ receptor 1	0.96	1.97	1.39	
Spp1, OSP	[GenBank:NM_012881]	Secreted phosphoprotein 1	0.97	4.21	1.98	
Vegfa	[GenBank:NM_031836]	Vascular endothelial growth factor A	1.11	1.80	1.43	
		*ATPase activity*				
Tap1, Cim, Abcb2	[GenBank:NM_032055]	Transporter 1, ATP-binding cassette, subfamily B (MDR/TAP)	1.22	1.91	2.24	
Tap2, Cim, Abcb3	[GenBank:NM_032056]	Transporter 2, ATP-binding cassette, subfamily B (MDR/TAP)	1.01	2.11	1.91	
		*G-protein coupled receptor binding*				
Ramp1	[GenBank:NM_031645]	Receptor (calcitonin) activity modifying protein 1	2.16	0.53	0.93	
Ramp2	[GenBank:NM_031646]	Receptor (calcitonin) activity modifying protein 2	0.88	1.57	1.31	

^a^Genes separated into molecular function subcategories represented in the list of gene changing at least 1.45-fold by TNFα as either mitogen-activated kinase kinase (MEK)/extracellular regulated kinase (ERK) dependent or MEK/ERK independent. Specifically defined subcategories were identified in the molecular function gene ontology as significantly represented (*P *< 0.01) within the MEK/ERK dependent or MEK/ERK independent lists.

**Figure 3:**
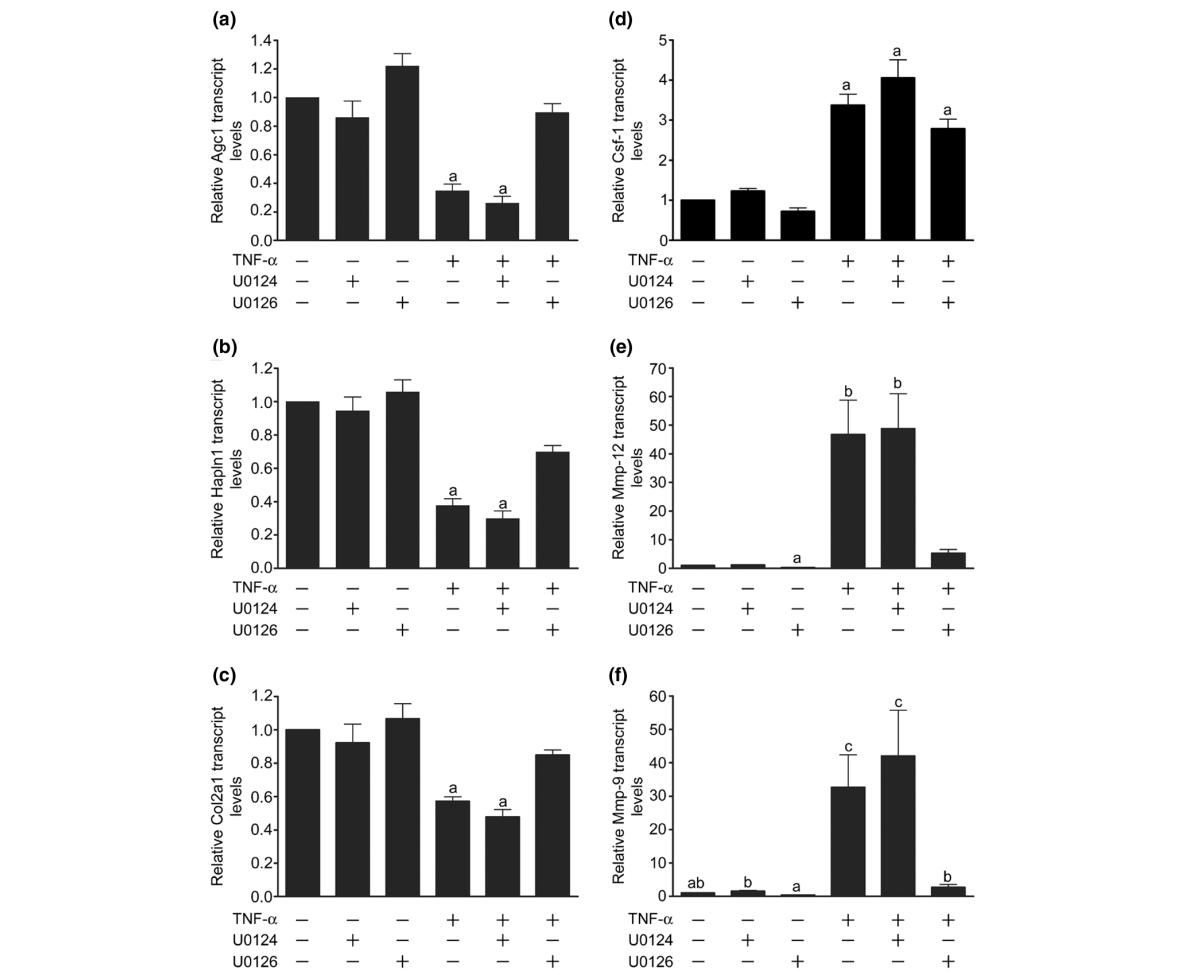
**TNFα regulates cartilage-selective matrix genes and proteinases in a MEK1/2-dependent manner**. Chondrocytes were pretreated with dimethyl sulfoxide, U0124 (10 μM) or U0126 (10 μM) for 30 minutes prior to treatment with TNFα for 24 hours. Total mRNA was collected and analysed for **(a) **aggrecan (Agc1), **(b) **link protein (Hapln1), **(c) **type II collagen (Col2a1), **(d) **macrophage colony-stimulating factor 1 (Csf-1), **(e) **matrix metalloproteinase-12 (Mmp-12) and **(f) **matrix metalloproteinase-9 (Mmp-9), and 18S transcript levels by quantitative real-time PCR. (a) to (f) Data were analysed by the ΔΔCT method to acquire matrix gene transcript levels relative to 18S transcript levels and were normalized to DMSO-treated cells. Data were log-transformed prior to analysis by one-way analysis of variance followed by Tukey's post-hoc tests. Unlabelled bars or bars labelled with the same lowercase letter are not significantly different (*P *> 0.05). Data are expressed as the mean ± standard error of five independent experiments – except (c), four independent experiments. MEK, mitogen-activated kinase kinase.

To validate the changes in gene expression in response to TNFα-induced MEK/ERK signalling determined by the microarray analysis, we identified the relative changes in transcript levels of the extracellular matrix components Agc1, Hapln1, and Col2a1, proteases Mmp-9 and Mmp-12, as well as the inflammatory cytokine macrophage Csf-1 (Figure 3). TNFα decreased Agc1 and Hapln1 (Figure 3a, b) and increased Mmp-9 and Mmp-12 (Figure 3e, f) in a MEK/ERK-dependent manner. In addition, Col2a1 – a gene not identified as MEK/ERK sensitive by microarray analysis – was also determined to be MEK/ERK sensitive (Figure 3c). Pretreatment with U0126, however, only partially attenuated the TNFα-induced reductions in Agc1, Hapln1 and Col2a1 transcript levels – to a level only moderately, but not significantly, lower than control treated cultures, suggesting the possible involvement of other pathways (Figure 3a to 3c). Conversely, TNFα-induced increases in macrophage Csf-1 were independent of MEK/ERK signalling (Figure 3d). As anticipated, the inactive U0126 analogue U0124 had no effect in any of the assays tested. Taken together, these results suggest that U0126 may attenuate the changes in chondrocyte gene expression towards a catabolic phenotype while allowing for inflammatory processes to be undisturbed.

### Regulation of Sox9 and NFκB activity by TNFα are independent of MEK/ERK signalling

**Figure 4:**
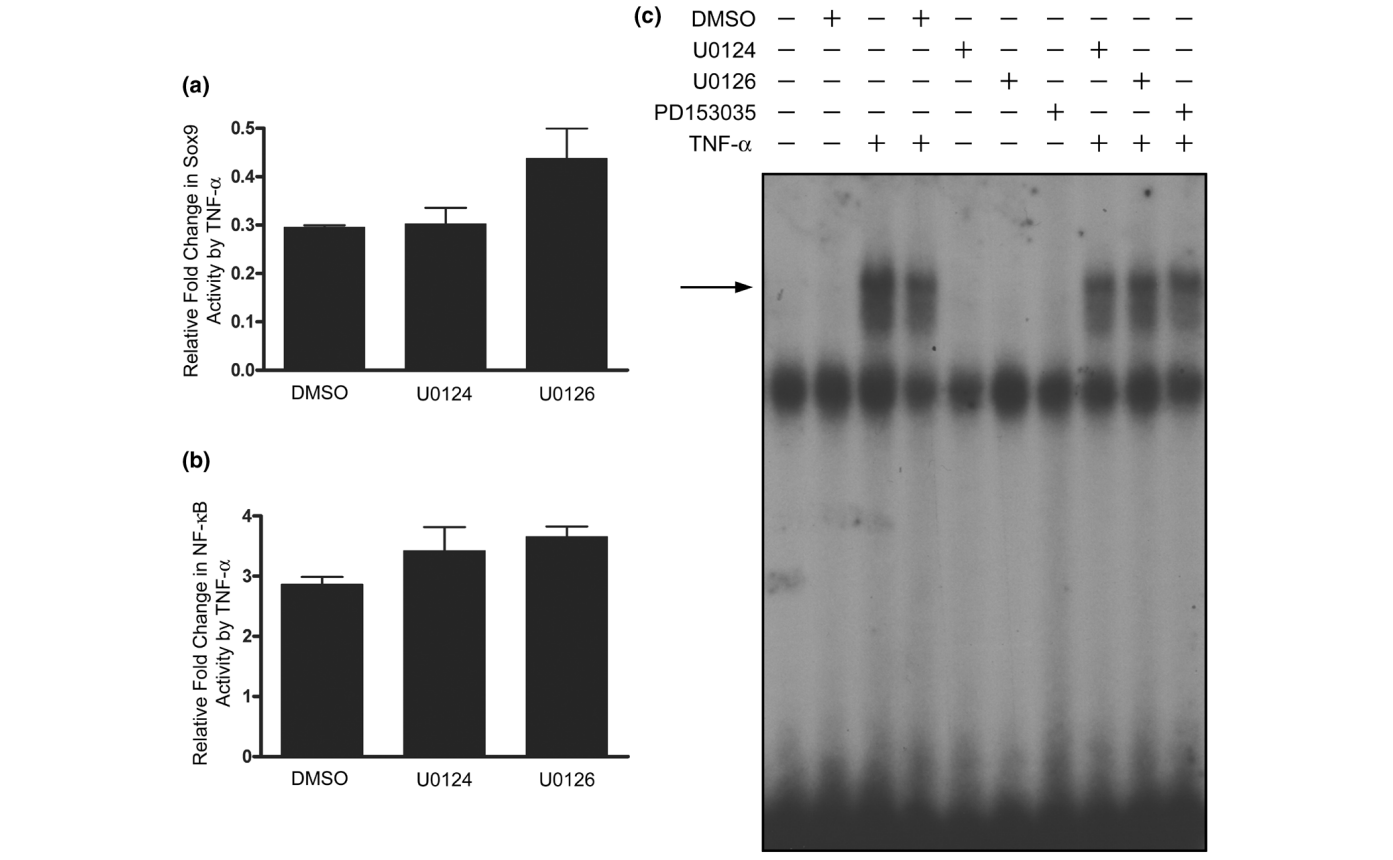
**TNFα-induced changes to Sox9 and NFκB functional activity are independent of MEK1/2 activity**. Chondrocytes transfected with **(a) **Sox9 or **(b) **NFκB reporters were pretreated with dimethyl sulfoxide (DMSO), U0124 (10 μM) or U0126 (10 μM) for 30 minutes followed by treatment with TNFα (30 ng/ml) for 24 hours. Data are ratios of (a) Sox9-regulated or (b) NFκB-regulated firefly luciferase units to constitutive cytomegalovirus-regulated renilla luciferase units in TNFα-treated cultures normalized to their respective DMSO-treated, U0124-treated or U0126 control-treated cultures. Data were log-transformed prior to analysis by paired *t *tests to determine significant reporter regulation by TNFα, followed by one-way analysis of variance to determine significant differences between the effects of DMSO, U0124 or U0126 pretreatment on TNFα-regulated reporter activity. Data are expressed as the mean ± standard error of four independent experiments. **(c) **Cells were pretreated with vehicle, DMSO, U0124 (10 μM) or U0126 (10 μM), or PD153035 (1 μM) for 30 minutes followed by treatment with TNFα (30 ng/ml) for 24 hours. Nuclear extracts (10 μg) were incubated with ^32^P-radiolabelled κB-consensus DNA. Resulting protein-DNA complexes were resolved on 4% polyacrylamide gels and exposed by autoradiography. Arrow, NFκB p65-containing protein-DNA complexes, as previously described [12]. The autoradiograph displayed is representative of three independent experiments.

We next wanted to determine the possible molecular basis for TNFα-modulated, U0126-sensitive gene expression. First, we investigated whether U0126 affected the ability of TNFα to regulate the activity of the transcription factors Sox9 and NFκB, which are known to be regulated by TNFα in chondrocytes [10,12]. As expected, TNFα significantly reduced the level of Sox9 activity and increased the level of NFκB activity in chondrocytes (*P *< 0.01; Figure 4a, b). There was no significant effect, however, on the level of inhibition or the induction of Sox9 and NFκB activity, respectively, by either U0124 or U0126 (*P *> 0.05; Figure 4a, b). Furthermore, we found that TNFα-induced DNA binding of NFκB was reduced by pretreatment with DMSO (vehicle for the inhibitors) and was not further reduced by pretreatment with U0124, U0126 or the selective epidermal growth factor receptor inhibitor, PD153035 (Figure 4c). These results indicate that transcription factors other than Sox9 and NFκB are targets of TNFα-induced MEK/ERK signalling.

### Egr-1 DNA binding is increased in a TNFα-induced MEK/ERK-dependent manner

To determine additional, candidate transcription factors that may regulated by MEK/ERK, we considered that Egr-1 is a known early target of MEK/ERK signalling and that IL-1 induction of Egr-1 inhibits the activity of the human type II collagen proximal promoter [31]. We therefore focused the remainder of our study on Egr-1 and its possible role in regulating U0126-sensitive TNFα-induced genes.

**Figure 5:**
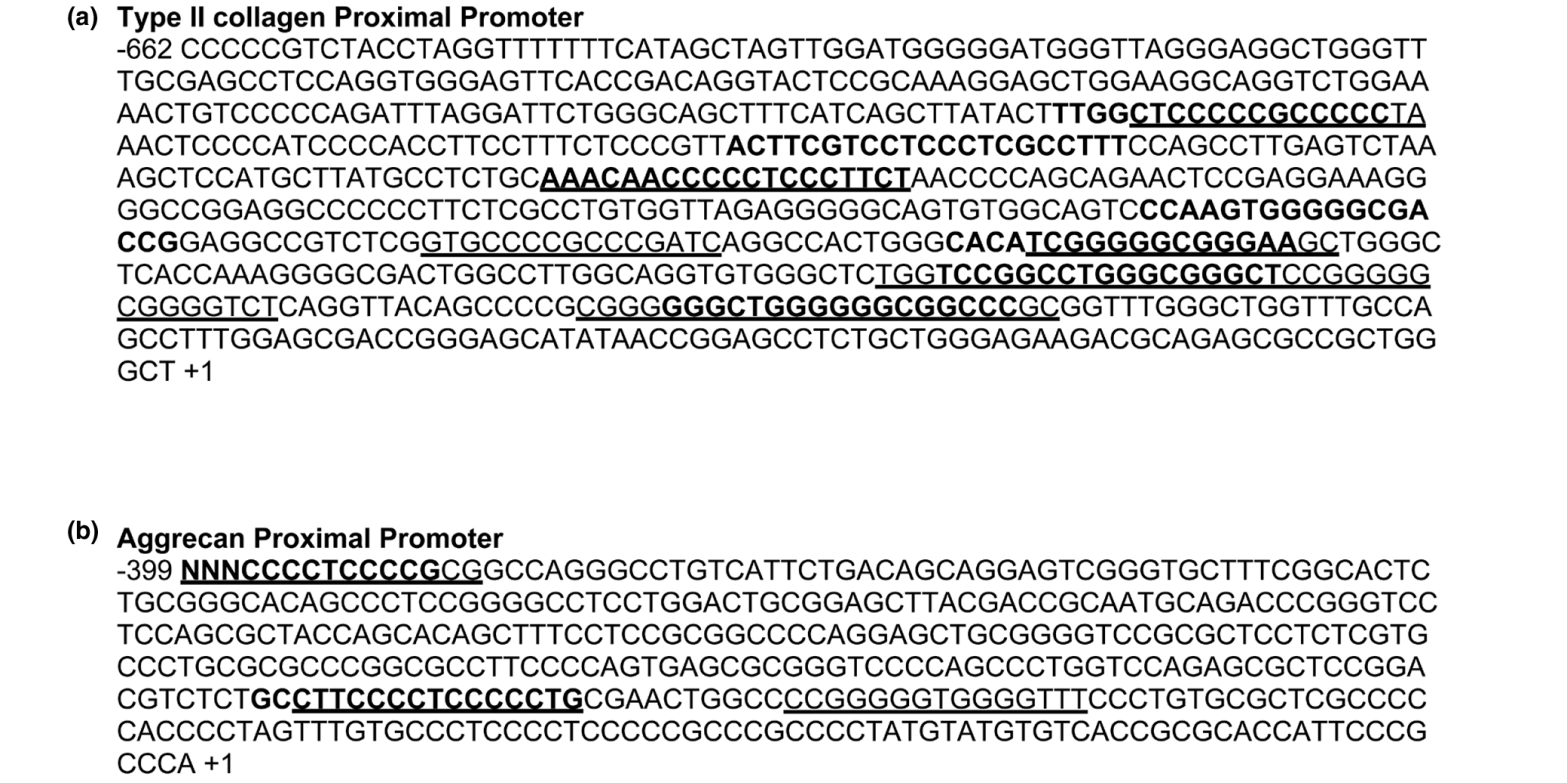
**Proximal promoters and overlapping binding regions for Sp1 and Egr-1**. Proximal promoters of rat type II collagen and aggrecan, but not of link protein, have overlapping binding regions for Sp1 and Egr-1. Upstream regions of **(a) **the rat type II collagen, **(b) **aggrecan and **(c) **link protein were analysed by TRANSFAC [26] for transcription factor binding sites. (a) and (b) Proximal to the transcriptional start site, the type II collagen and aggrecan promoters have multiple putative Sp1 (underlined) and Egr-1 binding sites (bold), some of which are found in overlapping regions.

We identified multiple putative Egr-1 binding sites in the promoter regions of the rat Col2a1 and Agc1 genes that were proximal to the transcription initiation site and overlapped with putative Sp1 binding sites (Figure 5). TNFα treatment of chondrocytes over 24 hours did not alter the Egr-1 protein levels, and neither did treatment for 90 minutes alter the nuclear localization of Egr-1 (data not shown).

**Figure 6:**
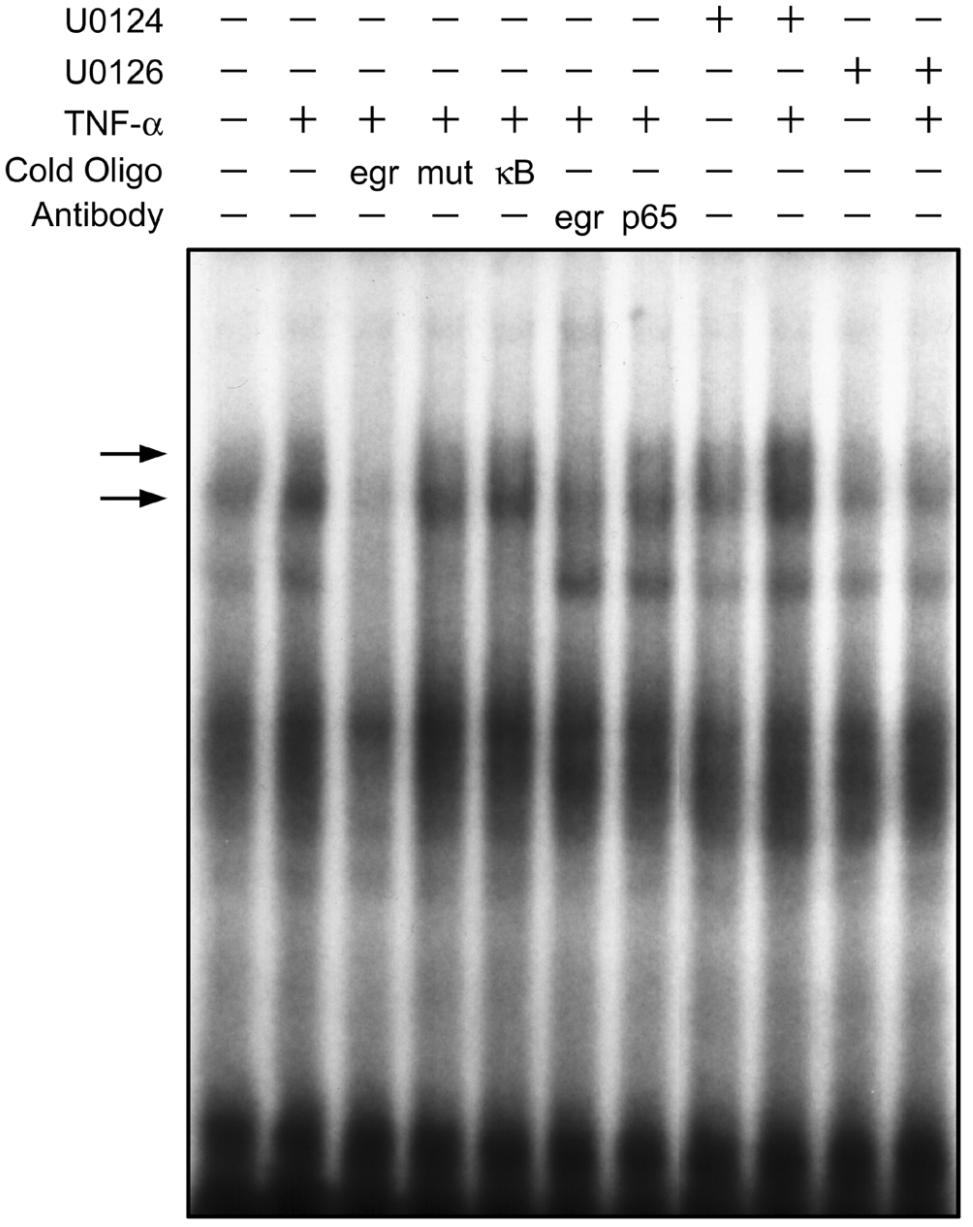
**Egr-1 DNA binding activity is increased by TNFα-induced MEK1/2 signalling in chondrocytes**. Cells were pretreated with dimethyl sulfoxide, U0124 (10 μM) or U0126 (10 μM) for 30 minutes prior to treatment with vehicle (-) or with 30 ng/ml TNFα (+) for 90 minutes. Nuclear extracts were incubated with ^32^P-radiolabelled oligodeoxynucleotides corresponding to the Egr consensus DNA binding sequence. In some cases, the nuclear extracts were incubated with 100-fold excess of cold specific Egr consensus oligodeoxynucleotides (egr), mutant Egr oligodeoxynucleotides (mut) or nonspecific oligodeoxynucleotides corresponding to the NFκB consensus sequence (κB). For antibody interference assays, nuclear extracts were preincubated with specific antibody for Egr-1 (egr) or nonspecific antibody for NFκB p65 isoform (p65). Resulting protein-DNA complexes were resolved on 4% polyacrylamide gels and exposed by autoradiography. Arrows, Egr-1-containing complexes. The autoradiograph shown is representative of three independent experiments.

We then used electrophoretic mobility shift assays to investigate whether the binding of Egr-1 to DNA was dependent on TNFα-induced MEK/ERK signalling. Nuclear extracts from chondrocytes treated with TNFα for 90 minutes increased the DNA binding of two complexes containing Egr-1 to an Egr consensus DNA binding site (Figure 6, arrowheads). Both complexes were reduced when extracts were preincubated with a 100-fold molar excess of double-stranded cold Egr consensus ODNs, but not with cold mutant Egr ODNs or NFκB consensus ODNs (Figure 6, arrowheads). Compared with preincubation of extracts with the anti-NFκB p65 antibody, preincubation of extracts with the anti-Egr-1 antibody specifically reduced the DNA-protein complexes attributed by the Egr consensus ODN competition studies to be a result of Egr/DNA binding (Figure 6, arrowheads). Pretreatment of cells with U0126 attenuated the increase in complex formation of both identified complexes. The binding of the identified complexes to DNA was inhibited by pretreatment with U0126 but not with U0124, indicating DNA binding of Egr-1 is dependent on TNFα-activated MEK/ERK signalling.

### Egr family DNA binding is responsible for decreased chondrocyte matrix gene expression

**Figure 7:**
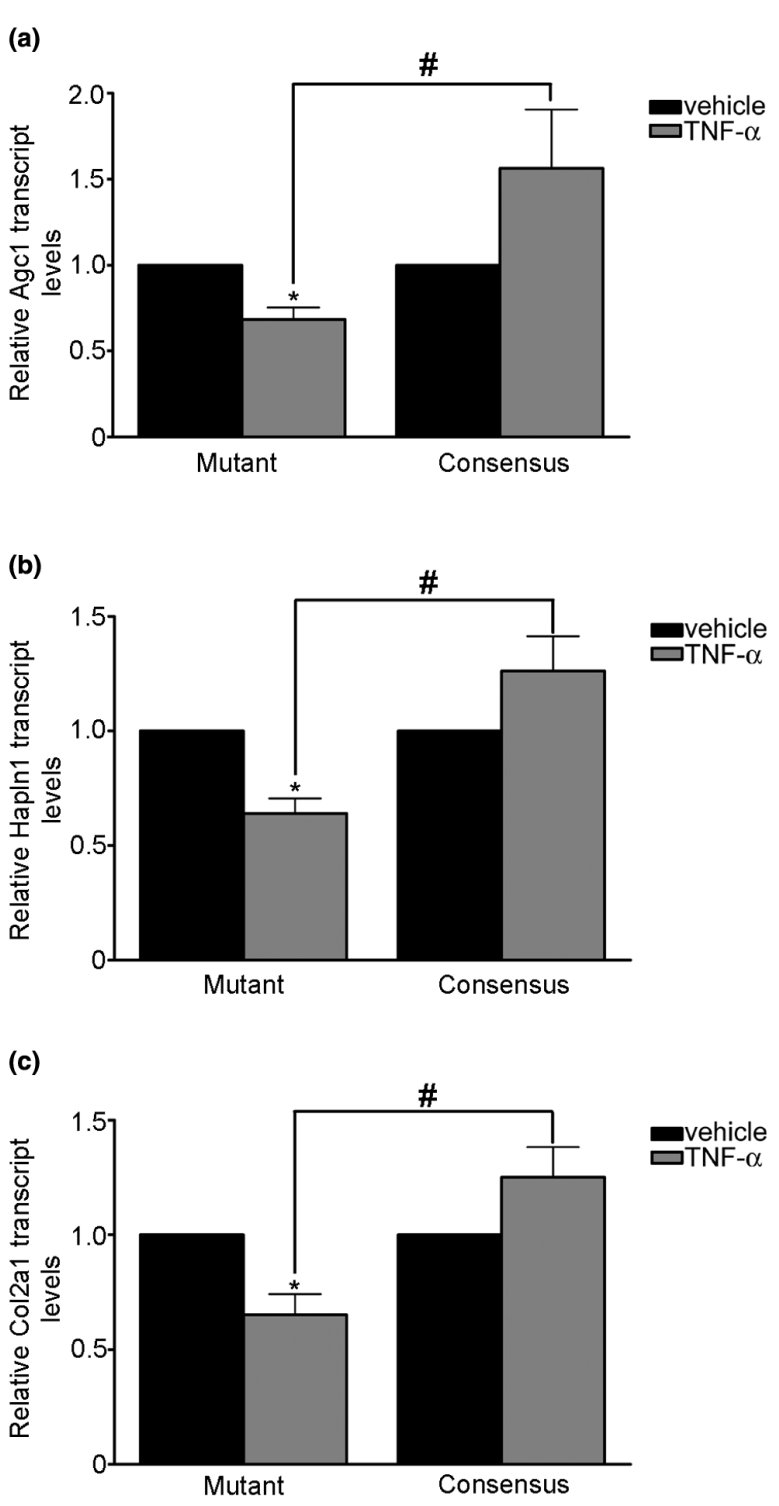
**Competitive inhibition of Egr transcription factor-DNA binding attenuates TNFα decreases in cartilage matrix transcripts**. Chondrocytes were transfected with 2 μM double-stranded, phosphorothiol-modified oligodeoxynucleotides containing Egr mutant or consensus DNA binding sequences and were treated with vehicle (black bars) or TNFα (grey bars) for 24 hours. Total RNA was collected and analysed by quantitative real-time PCR for **(a) **Agc1, **(b) **Hapln1 and **(c) **Col2a1, or 18S transcript levels. Data were analysed by the ΔΔCt method to acquire matrix gene transcript levels relative to 18S. Data from cells transfected with the Egr mutant or consensus oligodeoxynucleotides were normalized to vehicle-treated cultures and were log-transformed prior to analysis by paired and Student's *t *tests. *Significant difference (*P *< 0.05 by paired *t *test) in transcript levels compared with vehicle-treated cells transfected with the same oligodeoxynucleotide. ^#^Significant difference in transcript levels between cultures transfected with mutant or consensus Egr binding sequences and treated with TNFα (*P *< 0.05 by Student's *t *test). Results are displayed as the mean ± standard error of five independent experiments.

To determine whether decreases in chondrocyte selective matrix gene expression in response to TNFα were dependent on the genomic DNA binding activity of Egr family members, we transfected cells with double-stranded ODNs containing phosphorothiolate modifications corresponding to the cognate and a mutated form of the Egr-DNA binding sequence (Figure 7). Transfection of cells with mutant double-stranded ODNs did not disrupt decreases induced by TNFα to Col2a1, Agc1 or Hapln1 transcript levels. Transfection using the cognate Egr double-stranded ODNs, however, attenuated the decreases in transcript levels of Col2a1, Agc1 and Hapln1 by TNFα. Egr-containing complexes, probably that include Egr-1, are therefore responsible for the reduced transcript levels of cartilage selective matrix genes in response to TNFα in chondrocytes.

## Discussion

In the present study, we used the MEK1/2 inhibitor U0126 to identify the possible contribution of the MEK/ERK signalling pathway to changes in chondrocyte gene expression in response to TNFα. Inspection of the ~20% of TNFα-regulated chondrocyte mRNAs whose expression was modulated by MEK1/2 revealed a significant representation of genes whose protein products localized to the extracellular space, and had proteinase activity (for example, Mmp-9 and Mmp-12, which were induced by TNFα) or hyaluronic acid binding activity (for example, the matrix-associated genes Agc1 and Hapln1, which were suppressed by TNFα). Mmp-9 and Mmp-12 cleave selective proteoglycans and collagens [32-34] while Mmp-9 is also an important mediator of inflammatory arthritis [35]. Furthermore, we have shown that increases in transcripts encoding proinflammatory genes, such as macrophage Csf-1, were U0126 insensitive. Collectively these results suggest the intriguing notion that, compared with the TNFα-regulated transcript levels of genes involved in inflammation, TNFα-induced matrix catabolism may selectively require MEK/ERK. Further efforts will be required to assess whether similar mechanisms might operate in adult rat or human chondrocytes, or in cells isolated from patients with arthritis. Nonetheless, our data – for the first time – suggest that MEK inhibitors modify the excessive matrix degradation in arthritis.

Consistent with TNFα-induced increases in macrophage Csf-1 transcript levels observed in this study, macrophage Csf-1 protein levels are also induced by TNFα in chondrocytes [36]. In rat articular chondrocytes, macrophage Csf-1-induced signalling increases its own expression and the expression of the matricellular protein CCN2 (formerly known as connective tissue growth factor) [37]. CCN2 is required for Col2a1 and Agc1 expression in mouse chondrocytes [38] yet does not result in hypertrophic differentiation of rat articular chondrocytes [39]. Taken together, inhibition of TNFα-induced MEK/ERK or downstream transcription factors may rescue cartilage ECM gene expression and promote articular cartilage regeneration through continued macrophage Csf-1 expression.

In immortalized chondrocytes, NFκB-DNA binding activity is dependent on TNFα-induced MEK/ERK signalling [10], consistent with studies in other immortalized cells such as B-cell lymphoma cell lines [40]. In our present study using primary chondroctyes, however, both TNFα-regulated NFκB reporter activity and NFκB-DNA binding were unaltered by MEK/ERK inhibition. Immortalized cells may therefore have altered signalling that activates NFκB in a MEK/ERK-dependent manner by TNFα. Furthermore, we showed that pretreatment of primary chondrocytes with DMSO or DMSO-soluble inhibitors, such as U0124, U0126 and PD153035, reduced TNFα-activated NFκB-DNA binding activity. The regulation of NFκB-DNA binding in primary cells can therefore be explained by the nonspecific effect of DMSO on NFκB activation.

In the present study we determined that, in addition to NFκB, TNFα-regulated reductions in Sox9 activity were also independent of MEK/ERK signalling. Previous studies from our laboratory have shown that reductions in Sox9 activity by TNFα are dependent on NFκB nuclear translocation [10,12], a mechanism probably involving reductions in p300 histone acetylase activity associated with Sox9 [12]. MEK/ERK-independent reductions in Sox9 activity could therefore explain the inability of U0126 to completely reverse the TNFα-induced reductions in cartilage ECM gene transcript levels observed in this study.

We showed that Egr-1 DNA binding was increased by TNFα in a U0126-sensitive fashion. Moreover, competitive inhibition of Egr-1 binding to genomic targets attenuated decreases in cartilage ECM genes in response to TNFα. These results suggest that TNFα can modify gene expression in chondrocytes via MEK/ERK through the induction of Egr-1 DNA binding activity. Treatment of chondrocytes with IL-1 increases the Egr-1 protein and DNA binding, leading to decreased human type II collagen promoter activity through competition of Egr-1 for the Sp1 binding sites [31]. Previous studies have also identified that there are putative Sp1 binding sites in the aggrecan promoter of the chick, mouse and rat [25,41]. In this study, we identified putative overlapping binding sites for Sp1 and Egr-1 in both the rat COL2A1 and AGC1 promoters proximal to the transcriptional start site. Although beyond the scope of our current report, Col2a1 and Agc1 transcription are probably regulated by inhibitory actions of Egr-1 in competition for Sp1 binding sites. Collectively, these data suggest that, in chondrocytes, alterations in Egr-1 DNA binding activity by TNFα-induced MEK/ERK signalling is necessary for the transcriptional regulation of downstream cartilage ECM genes.

In the current study, pharmacological inhibition of MEK resulted in significant attenuation of the TNFα-induced decreases to Col2a1, Agc1 and Hapln1 24 hours post treatment. Depending on the species the half-life of Col2a1 mRNA in chondrocytes is between 15 and 18 hours [27,29,30], whereas the half-life of Agc1 mRNA is about 4 hours in bovine articular chondrocytes [28]. In this study we observed ~50% reduction in Col2a1 and ~70% reduction in Agc1 transcript levels after 24 hours. Previous studies from our laboratory have indicated that inhibition of Col2a1 transcripts in response to TNFα results from an inhibition of transcription and not from changes to message stability [10]. Furthermore, treatment of chondrocytes with actinomycin D, a transcription inhibitor, decreased Col2a1 and Agc1 mRNAs to a level comparable with that of TNFα treatment alone (unpublished data). Collectively, TNFα-induced reductions in cartilage ECM transcripts in this study are consistent with regulation of these mRNAs through inhibition of transcription. Although it is possible that TNFα may modulate cartilage ECM transcript expression in an indirect fashion, the relatively delayed kinetics of TNFα-modulated cartilage ECM transcripts is probably due to the stability of the mRNAs.

## Conclusion

Most therapies for rheumatoid arthritis, specifically biologics, are targeted towards TNFα protein and not towards its activated signalling pathways [42]. Targeted therapies that block specific subcellular molecules involved in TNFα-activated signalling pathways, however, may be useful in selectively modifying chondrocyte responses to TNFα. Our data suggest that MEK/ERK may selectively be required for TNFα-modulated proteinase and cartilage ECM transcripts, but not for inflammatory gene transcripts. These results raise the intriguing notion that MEK/ERK inhibitors might be used to block the ability of TNFα to promote matrix catabolism but leave perhaps more beneficial effects of TNFα unaltered. In the long term, our observations may be of relevance for developing new methods of treating arthritis. In particular, antagonizing MEK/ERK or activating Egr-1 may be useful methodologies for reversing cartilage degradation observed in both osteoarthritis and rheumatoid arthritis.

## Abbreviations

Agc1: aggrecan 1; Col2a1: type II collagen (α); Csf-1: colony stimulating factor 1; DMSO: dimethyl sulfoxide; ECM: extracellular matrix; Egr: early growth response; ERK: extracellular regulated kinase; IFN: interferon; IL: interleukin; MEK: mitogen-activated kinase kinase; Mmp: matrix metalloproteinase; NF: nuclear factor; ODN: oligodeoxynucleotide; PCR: polymerase chain reaction; Sox: Sry-type high-mobility group box; TBST: Tris-buffered saline with Tween-20; TNF: tumour necrosis factor.

## Competing interests

The authors declare that they have no competing interests.

## Authors' contributions

JSR carried out all aspects of the study, including the initial design of the study, microarray analysis, immunoblotting, electrophoretic mobility shift assay, quantitative real-time PCR and transfection studies, drafting and editing of the manuscript, and preparation of the figures. SMB was involved with the design and coordination of the study. AL was involved with the design and coordination of the study, drafting and editing of the manuscript.
